# Genome-Wide Comparative Analysis of Heat Shock Transcription Factors Provides Novel Insights for Evolutionary History and Expression Characterization in Cotton Diploid and Tetraploid Genomes

**DOI:** 10.3389/fgene.2021.658847

**Published:** 2021-06-08

**Authors:** Yajun Liang, Junduo Wang, Juyun Zheng, Zhaolong Gong, Zhiqiang Li, Xiantao Ai, Xueyuan Li, Quanjia Chen

**Affiliations:** ^1^Xinjiang Academy of Agricultural Science, Urumqi, China; ^2^Engineering Research Centre of Cotton of Ministry of Education, Xinjiang Agricultural University, Urumqi, China; ^3^Adsen Biotechnology Corporation, Urumqi, China

**Keywords:** genome-wide, heat shock transcription factor, *Gossypium* lineage, tandem duplication, expression divergence

## Abstract

Heat shock transcription factors (HSFs) are involved in environmental stress response and plant development, such as heat stress and flowering development. According to the structural characteristics of the HSF gene family, HSF genes were classified into three major types (HSFA, HSFB, and HSFC) in plants. Using conserved domains of HSF genes, we identified 621 HSF genes among 13 cotton genomes, consisting of eight diploid and five tetraploid genomes. Phylogenetic analysis indicated that HSF genes among 13 cotton genomes were grouped into two different clusters: one cluster contained all HSF genes of HSFA and HSFC, and the other cluster contained all HSF genes of HSFB. Comparative analysis of HSF genes in *Arabidopsis thaliana*, *Gossypium herbaceum* (A1), *Gossypium arboreum* (A2), *Gossypium raimondii* (D5), and *Gossypium hirsutum* (AD1) genomes demonstrated that four HSF genes were inherited from a common ancestor, A0, of all existing cotton A genomes. Members of the HSF gene family in *G. herbaceum* (A1) genome indicated a significant loss compared with those in *G. arboretum* (A2) and *G. hirsutum* (AD1) A genomes. However, HSF genes in *G. raimondii* (D5) showed relative loss compared with those in *G. hirsutum* (AD1) D genome. Analysis of tandem duplication (TD) events of HSF genes revealed that protein-coding genes among different cotton genomes have experienced TD events, but only the two-gene tandem array was detected in *Gossypium thurberi* (D1) genome. The expression analysis of HSF genes in *G. hirsutum* (AD1) and *Gossypium barbadense* (AD2) genomes indicated that the expressed HSF genes were divided into two different groups, respectively, and the expressed HSF orthologous genes between the two genomes showed totally different expression patterns despite the implementation of the same abiotic stresses. This work will provide novel insights for the study of evolutionary history and expression characterization of HSF genes in different cotton genomes and a widespread application model for the study of HSF gene families in plants.

## Background

Cotton (genus *Gossypium*) is one of the most important economic crops worldwide, which provides the major resource of natural fiber for human beings over the past decades. The genus *Gossypium* contains over 45 diploid (designated as A to G and K) and 7 tetraploid (designated as AD1 to AD7) species (Paterson et al., 2012; Grover et al., 2015; Gallagher et al., 2017). To facilitate the genetic breeding and crop improvement of cotton, various cotton genomes were released for the community, such as eight diploid genomes: *Gossypium herbaceum* (A1) (Huang et al., 2020), *Gossypium arboreum* (A2) (Li et al., 2014), *Gossypium thurberi* (D1) (Grover et al., 2019), *Gossypium raimondii* (D5) (Wang K. et al., 2012), *Gossypium turneri* (D10) (Udall et al., 2019a), *Gossypium longicalyx* (F1) (Grover et al., 2020), *Gossypium australe* (G2) (Cai et al., 2020), and *Gossypioides kirkii* (K) (Udall et al., 2019b), and five tetraploid genomes: *Gossypium hirsutum* (AD1) (Li et al., 2015), *Gossypium barbadense* (AD2) (Liu et al., 2015), *Gossypium tomentosum* (AD3) (Chen et al., 2020), *Gossypium mustelinum* (AD4) (Chen et al., 2020), and *Gossypium darwinii* (AD5) (Chen et al., 2020). Previous reports illustrated that the ancestor of cotton A0 and D5 genome species diverged from a common ancestor with *Bombax ceiba* at approximately 24 million years ago (Mya). Subsequently, cotton A0 and D5 genome species diverged from their ancestor at approximately 4.8 Mya. After the split of their ancestor, cotton A0 and D5 genome species crossed and generated cotton allotetraploid AD genome species at approximately 1.6 Mya (Huang et al., 2020). Later, the ancestor of allotetraploid cotton has diverged into *G. hirsutum* (AD1) and *G. barbadense* (AD2) (Liu et al., 2015). Less than 1 Mya (∼0.7 Mya), the ancestor of cotton A1 and A2 genome species experienced divergence and generated cotton diploid species, *G. herbaceum* (A1) and *G. arboreum* (A2) (Huang et al., 2020). A clear evolutionary route and available whole-genome sequences in cotton genomes provide an excellent opportunity for studying the evolutionary history and functional characteristics of genes or gene families related to critical phenotypes or traits in cotton. However, several important mechanisms, including tandem duplication (TD) event, have been illustrated clearly. Previous reports mentioned that a TD event is a critical mechanism in plants which brings an increase in the number of gene copies leading to the expansion of the gene family (Graham, 1995).

Heat shock transcription factors (HSFs) serve as the major activators of heat shock proteins (HSPs) with the help of binding to the promoter regions of HSP genes in plants. This enables HSP genes to regulate transcription in response to heat stress for adapting to various environmental dynamic changes (Ahuja et al., 2010; Guo et al., 2016). HSF proteins contain several conserved domains or motifs, including DNA-binding domain (DBD), oligomerization domain (OD or HR-A/B), nuclear localization signals (NLS), nuclear export signals (NES), activator peptide proteins (AHA), and repressor domains (RD) (Scharf et al., 2012; Li et al., 2019). The DBD is the major core domain of HSF protein, which is responsible for binding to the heat shock elements (HSEs) of HSF genes and performs by regulating the transaction activity of HSF genes (Scharf et al., 2012). The OD (or HR-A/B) includes two hydrophobic heptad repeats. Based on the length of a flexible linker between DBD and OD and the amino acids of OD regions, members of the HSF gene family can be divided into three major types, namely HSFA, HSFB, and HSFC (Zhu et al., 2017; Chen et al., 2018). Until now, the identification of the HSF gene family has been investigated in several genome-sequenced plant species, such as *Solanum lycopersicum* (24) (Doring et al., 2000), *Arabidopsis thaliana* (21) (Guo et al., 2008), *Oryza sativa* (25) (Scharf et al., 2012), *Zea mays* (25) (Lin et al., 2011), *Glycine max* (26) (Chung et al., 2013), *Brassica oleracea* (35) (Lohani et al., 2019), *Brassica rapa* (36) (Lohani et al., 2019), *Brassica napus* (64) (Lohani et al., 2019), *Sesamum indicum* (30) (Dossa et al., 2016), *Prunus mume* (18) (Wan et al., 2019), *Fagopyrum tataricum* (29) (Liu et al., 2019), *Cicer arietinum* (20) (Zafar et al., 2016), *Vitis vinifera* (19) (Guotian et al., 2018), *Capsicum annuum* (25) (Guo et al., 2015), and *Brassica juncea* (60) (Li et al., 2020). For these plant species, the HSF gene family was classified and investigated based on whole genomes and an expression analysis of the members of the HSF gene family under different experimental designs was conducted. Wang et al. reported 40 HSF genes in *G. hirsutum* (AD1) D genome using EST assembly and genome-wide analyses (Wang et al., 2014).

In this project, we performed genome-wide comparative genomics analysis for the HSF gene family among 13 cotton genomes. Based on the evolutionary relationship among *G. herbaceum* (A1), *G. arboreum* (A2), *G. raimondii* (D5), and *G. hirsutum* (AD1), we detected the formation of the HSF gene family and traced the evolution of HSF genes in *G. hirsutum* (AD1) genome. According to the genome-wide analysis of TD events, we investigated the influence of TD events on the generation of HSF genes among 13 cotton genomes. However, we used the expression analysis to reveal the functional differences of HSF genes under the same abiotic stresses in *G. hirsutum* (AD1) acc. TM-1 and *G. barbadense* (AD2) acc. Hai7124. This project will provide novel insights for understanding the evolutionary history and expression characterization of the HSF gene family, paving the way for genetic breeding and molecular improvement of cotton diploid and tetraploid species.

## Materials and Methods

### Data Resources

The latest genomic data of cotton diploid genomes, *G. herbaceum* (A1, WHU_v1), *G. arboreum* (A2, WHU_v1), *G. thurberi* (D1, ISU_v1), *G. raimondii* (D5, NSF_v1), *G. turneri* (D10, NSF_v1_a2), *G. longicalyx* (F1, NSF_v1), *G. australe* (G2, CRI_v1.1), and *G. kirkii* (K, ISU_v3_a3), and tetraploid genomes, *G. hirsutum* (AD1, WHU_v1), *G. barbadense* (AD2, HGS_v1.1), *G. tomentosum* (AD3, HGS_v1.1), *G. mustelinum* (AD4, JGI_v1.1), and *G. darwinii* (AD5, HGS_v1.1), are downloaded from CottonGen^1^ (Yu et al., 2014). The *A. thaliana* TAIR11 genome data is downloaded from TAIR^2^ (Lamesch et al., 2012). The profile hidden Markov models (HMMs) of HSF_DNA-bind (or DBD) domain (PF00447.18) is downloaded from Pfam 33.1 (May 2020, 18,259 entries)^3^ (Bateman et al., 2004). The RNA-seq expression data of *G. hirsutum* (AD1) acc. TM-1 and *G. barbadense* (AD2) acc. Hai7124 are retrieved from the Sequence Read Archive (SRA) database with accession number PRJNA490626 (Kodama et al., 2012).

### Identification of HSF Transcription Factors

Firstly, the putative HSFs of 13 cotton diploid and tetraploid genomes were identified by HMMER (v3.2.1-foss-2018b) program with “trusted cutoff” as threshold (Potter et al., 2018). All protein sequences of putative HSFs were used to perform multiple sequence alignments (MSA) through ClustalW2 with the protein weight matrix Gonnet (Larkin et al., 2007). Secondly, the higher conserved HSF protein sequences were used to construct species-specific profile HMMs of HSF domains among different species by using the “hmmbuild” module in HMMER (v3.2.1-foss-2018b). Using the latest profile HMMs of HSFs, the target genes were identified from 13 different cotton diploid and tetraploid genomes with HMMER (v3.2.1-foss-2018b). Finally, InterProScan was used to investigate the validation of HSFs among different cotton genomes (Jones et al., 2014). Additionally, the oligomerization domain (OD or HR-A/B) was examined by the MARCOIL database (Zimmermann et al., 2018).

### Phylogeny of the HSF Gene Family

ClustalW2 was employed to perform MSA with protein sequences of target HSFs from 13 different cotton species. Then, the MSA file with “meg” format were used to construct the phylogenetic tree through MEGA 7 with maximum likelihood (ML) statistical method and 1,000 bootstrap replications (Kumar et al., 2016). The phylogeny analysis of HSFs in *A. thaliana* and *Gossypium* species followed the same procedures.

### Analysis of Tandem Duplication Event

Following the identification of tandemly duplicated genes in PTGBase, all-against-all BLAST of protein sequences in 13 cotton species was employed to identify the paralogous gene pairs within single cotton species with E-value cut-off ≤1e-20 (Yu et al., 2015). After confirmation of paralogous gene pairs with high similarity anchored on closer location of identical genomic regions, tandemly duplicated genes were obtained in different cotton species, and one unrelated gene was allowed within one tandem array.

### Analysis of Collinear Relationship

Orthologous gene pairs between *G. herbaceum* (A1), *G. arboreum* (A2), and *G. hirsutum* (AD1) A genomes, as well as between *G. raimondii* (D5) and *G. hirsutum* (AD1) D genomes, were detected by the MCscanX software and validated by phylogenetic analysis (Wang Y. et al., 2012). First, the MCscanX software was employed to identify orthologous regions with the parameters (MATCH_SIZE = 5 and E_VALUE = 1e-10) among the above three cotton genome pairs. Then, after extracting orthologous regions that contained HSF genes, HSF orthologous gene pairs were obtained. This step would get multiple HSF gene pairs with all-against-all relationship due to the high similarity between *G. herbaceum* (A1), *G. arboreum* (A2), and *G. hirsutum* (AD1) A genomes and between *G. raimondii* (D5) and *G. hirsutum* (AD1) D genomes. So, phylogenetic analysis of these four cottons and *A. thaliana* genomes was used to validate collinear relationships among cotton genomes. The collinear analysis of *G. hirsutum* (AD1) and *G. barbadense* (AD2) A genomes, as well as *G. hirsutum* (AD1) and *G. barbadense* (AD2) D genomes, follows the above procedures.

### Expression Analysis of HSF Genes

Using the available RNA-seq data of different samples from SRA in NCBI, all raw data of different samples were trimmed and cleaned by Trimmomatic (v0.39) (Bolger et al., 2014), and all clean short reads were mapped to *G. hirsutum* (AD1) and *G. barbadense* (AD2) reference genome sequences through HISAT (version 2.2.1) (Kim et al., 2015). The StringTie (version 2.0.6) was used to calculate the fragments per kilobase of exon model per million mapped fragments (FPKM) of mapped short reads to target genes or transcripts in different cotton genomes and FPKM values were normalized by log2 (Pertea et al., 2015). The hierarchical cluster was generated by Cluster 3.0 software and the heat map of gene expression was drawn by TreeView version 1.2.0 software (Eisen et al., 1998).

## Results

### Identification of HSF Genes in Cotton Genomes

With the development of genome sequencing technology, more and more cotton genome sequences were released for the community, which facilitate the analysis of key gene families within plant whole genomes. Totally, 13 latest cotton genomics data, consisting of eight diploid and five tetraploid genomes, were downloaded from CottonGen (see text footnote 1; Table 1; Yu et al., 2014). According to the conservation of HSF proteins in plants, HSF genes contain HSF_DNA-bind (or DBD) conserved domain, which plays a main function of HSF genes in plant species. Based on the profile HMMs of the HSF domain in the Pfam 33.1 database (Bateman et al., 2004), we used the HMMER v3.2.1 software to rebuild species-specific profile HMMs with protein sequences of cotton diploid and tetraploid genomes (Potter et al., 2018). Using their species-specific profile HMMs, candidate HSF genes among eight diploid and five tetraploid genomes were identified within whole genomes. After manual curation, we obtained four HSF genes in *G. herbaceum* (A1, WHU_v1), with 17 in *G. arboreum* (A2, WHU_v1), 29 in *G. thurberi* (D1, ISU_v1), 33 in *G. raimondii* (D5, NSF_v1), 36 in *G. turneri* (D10, NSF_v1_a2), 38 in *G. longicalyx* (F1, NSF_v1), 36 in *G. australe* (G2, CRI_v1.1), 29 in *G. kirkii* (K, ISU_v3_a3), 78 in *G. hirsutum* (AD1, WHU_v1), 78 in *G. barbadense* (AD2, HGS_v1.1), 84 in *G. tomentosum* (AD3, HGS_v1.1), 79 in *G. mustelinum* (AD4, JGI_v1.1), and 80 in *G. darwinii* (AD5, HGS_v1.1) genomes (Supplementary File 1). From comparisons of HSF genes among 13 cotton genomes, *G. herbaceum* (A1) has the least number of HSF genes and *G. australe* (G2) has the greatest number of HSF genes in cotton diploid genomes. Out of five cotton tetraploid genomes, *G. hirsutum* (AD1) and *G. barbadense* (AD2) have the least number of HSF genes, and *G. tomentosum* (AD3) has the greatest number of HSF genes.

**TABLE 1:** Summary of genomic data in 13 genome-sequenced cotton species.

Categories	Species	Karyotype	Version	Accession	Chromosomes and scaffolds	Numbers of predicted genes	Numbers of predicted CDSs	Numbers of predicted proteins	Number of HSF genes
Diploid genomes	*Gossypium herbaceum*	A1	WHU_v1	africanum “Mutema,” A1-0076	13 + 719	43,952	43,952	43,952	4
	*Gossypium arboreum*	A2	WHU_v1	SXY1	13 + 1,256	43,278	43,278	43,278	17
	*Gossypium thurberi*	D1	ISU_v1	D1-35	13	31,520	31,520	31,520	29
	*Gossypium raimondii*	D5	NSF_v1	D5-4	13	40,743	41,030	41,030	33
	*Gossypium turneri*	D10	NSF_v1_a2	D10-3	13	39,692	38,871	38,871	36
	*Gossypium longicalyx*	F1	NSF_v1	F1-1	13	38,378	40,181	40,181	38
	*Gossypium australe*	G2	CRI_v1.1	PA1801(G2-lz)	13 + 551	38,281	38,281	38,281	36
	*Gossypioides kirkii*	K	ISU_v3_a3	JFW-EA	12	36,680	36,680	36,680	29
Tetraploid genomes	*Gossypium hirsutum*	AD1	WHU_v1	line TM-1	26 + 316	74,350	74,350	74,350	78
	*Gossypium barbadense*	AD2	HGS_v1.1	Mar-79	26 + 4,748	74,651	108,363	108,363	78
	*Gossypium tomentosum*	AD3	HGS_v1.1	7179	26 + 293	78,281	112,713	112,713	84
	*Gossypium mustelinum*	AD4	JGI_v1.1	1408120	26 + 2,147	74,660	106,487	106,487	79
	*Gossypium darwinii*	AD5	HGS_v1.1	AD5-32	26 + 308	78,303	97,407	97,407	80

To identify accurately the members of the HSF gene family, we used the entire available protein sequences in different cotton genomes. After curation, we found that several cotton tetraploid genomes released multiple protein sequences for one gene due to an alternative splice in genomes. Using the profile HMMs of HSF_DNA-bind, we identified 120, 135, 122, and 106 HSF protein sequences in *G. barbadense* (AD2), *G. tomentosum* (AD3), *G. mustelinum* (AD4), and *G. darwinii* (AD5) genomes, respectively. Finally, we obtained 78 representative HSF genes in *G. barbadense* (AD2) genome, with 84, 79, and 80 HSF genes in *G. tomentosum* (AD3), *G. mustelinum* (AD4), and *G. darwinii* (AD5) genomes, respectively.

### Phylogeny of HSF Genes Among Different Cotton Genomes

**FIGURE 1:**
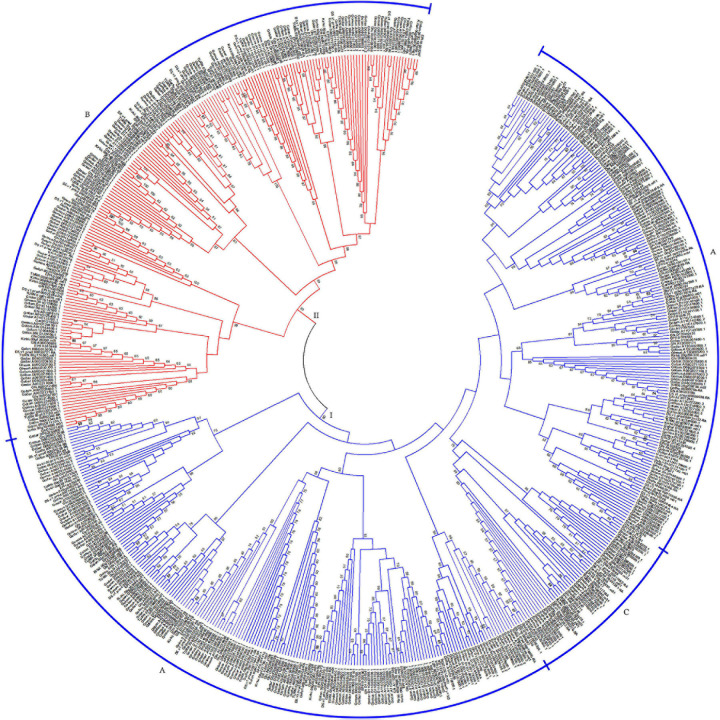
Phylogenetic relationship of HSF genes in 13 cotton genomes. The phylogenetic tree with maximum likelihood was constructed by MEGA software with 1,000 replications. I and II represent different groups that the HSF gene clustered. A, B, and C represent different types (HSFA, HSFB, and HSFC) of HSF genes in 13 cotton genomes.

Based on the characteristics of conserved domains of HSF genes, we obtained 621 target HSF genes among 13 cotton genomes. With protein sequences of the identified HSF genes, we constructed a phylogenetic tree to investigate the evolutionary relationship of all HSF genes among 13 cotton genomes. Following the phylogenetic tree, all HSF genes were grouped into two different clusters, including I and II clusters, among 13 cotton genomes (Figure 1). A previous report illustrated that the HSF gene family was classified into three different types, namely HSFA, HSFB, and HSFC, due to the number of amino acid residues between two heptad repeats (Nover et al., 2001). Based on the criteria of classification of HSF genes in plants, the I cluster contains 415 HSF genes representing 66.82% of the total HSF genes among 13 cotton genomes, which included 367 HSF genes in HSFA and 48 HSF genes in HSFC distributed into 13 diploid and tetraploid genomes. For the II cluster, 206 HSF genes were clustered together, which represented 33.18% of the total HSF genes in 13 cotton genomes, which belonged to the HSFB (Table 2).

**TABLE 2:** Different types of HSF genes in 13 cotton genomes.

Species	Karyotype	HSFA	HSFB	HSFC	Total HSFs
*G. herbaceum*	A1	2	1	1	4
*G. arboreum*	A2	6	8	3	17
*G. thurberi*	D1	19	8	2	29
*G. raimondii*	D5	18	12	3	33
*G. turneri*	D10	21	12	3	36
*G. longicalyx*	F1	23	13	2	38
*G. australe*	G2	22	12	2	36
*G. kirkii*	K	15	11	3	29
*G. hirsutum*	AD1	47	25	6	78
*G. barbadense*	AD2	47	25	6	78
*G. tomentosum*	AD3	50	28	6	84
*G. mustelinum*	AD4	49	25	5	79
*G. darwinii*	AD5	48	26	6	80
Total number		367	206	48	621

Upland cotton, *G. hirsutum* (AD1), was generated from hybridization between *G. raimondii* (D5) and the common progenitor, A0 genome, of *G. herbaceum* (A1) and *G. arboreum* (A2). However, out of four HSF genes in *G. herbaceum* (A1) genome, two HSF genes belonged to HSFA, with one HSF gene belonging to HSFB and one HSFC gene belonging to HSFC. For HSF genes in *G. arboreum* (A2) genome, six HSF genes were classified into HSFA, with eight HSF genes belonging to HSFB and three HSF genes belonging to HSFC. These HSF genes in *G. herbaceum* (A1) and *G. arboreum* (A2) originated from a common progenitor of all existing A genomes, including A genome in *G. hirsutum* (AD1). So, different types of HSF genes in *G. herbaceum* (A1) genome indicated a significant loss compared with *G. arboreum* (A2) and *G. hirsutum* (AD1) A genomes. However, different types of HSF genes in *G. raimondii* (D5) experienced relative loss compared with those in *G. hirsutum* (AD1) D genome.

### Comparative Analysis of HSF Genes in *A. thaliana* and Cotton Species

**FIGURE 2:**
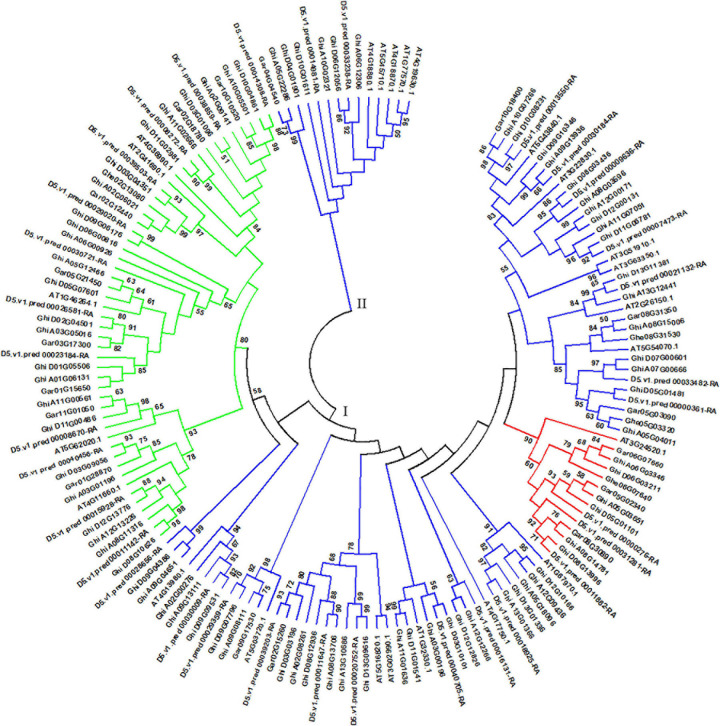
Phylogenetic analysis of HSF genes among *A. thaliana*, *G. herbaceum* (A1), *G. arboreum* (A2), *G. raimondii* (D5), and *G. hirsutum* (AD1) genomes. The phylogenetic tree with maximum likelihood was constructed by MEGA software with 1,000 replications. I and II represent different groups of HSF genes among the five plant species. Different types of HSF genes are shown by different colors. Blue, red, and green colors represent the HSFA, HSFB, and HSFC types of HSF genes, respectively.

*Arabidopsis thaliana* is an important model plant, and its genomic data were released more than 20 years, which brings opportunity for studying HSF genes in *A. thaliana* (Kaul et al., 2000). Based on the main conserved domain of HSF genes and *A. thaliana* TAIR11 genome, we identified 24 HSF genes in *A. thaliana* genome, which presents more HSF genes than the previous study of Guo et al. (2008). In this part, we collected 156 HSF genes in *A. thaliana*, *G. herbaceum* (A1), *G. arboreum* (A2), *G. raimondii* (D5), and *G. hirsutum* (AD1) genomes to perform comparative genomics analysis of HSF genes in *A. thaliana* and cotton genomes. Through phylogenetics analysis of HSF genes in *A. thaliana* and cotton genomes, all HSF genes among five species were clustered into two different groups, namely the I and II groups (Figure 2). In the I group, HSF genes that belonged to HSFA, HSFB, and HSFC were clustered together, and in the II group, the HSF genes all belonged to HSFA.

**FIGURE 3:**
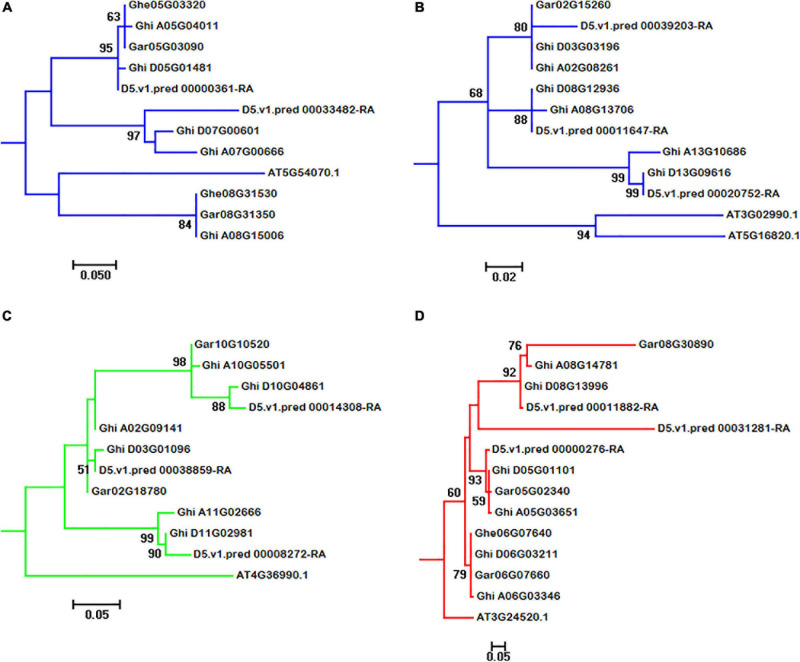
Subgroups of HSF genes in *A. thaliana*, *G. herbaceum* (A1), *G. arboreum* (A2), *G. raimondii* (D5), and *G. hirsutum* (AD1) genomes. **(A–D)** represent the subgroups of HSF orthologous genes in *A. thaliana*, *G. herbaceum* (A1), *G. arboreum* (A2), *G. raimondii* (D5), and *G. hirsutum* (AD1) genomes, respectively.

In *A. thaliana* genome, 18 HSF genes belonged to HSFA, with five belonging to HSFB and one belonging to HSFC. From the phylogeny of *A. thaliana* and cotton species, AT5G54070.1 (belonged to HSFA) has two orthologous genes (Ghe05G03320 and Ghe08G31530) in *G. herbaceum* (A1) genome, two orthologous genes (Gar05G03090 and Gar08G31350) in *G. arboreum* (A2) genome, two orthologous genes (D5.v1.pred_00000361-RA and D5.v1.pred_00033482-RA) in *G. raimondii* (D5) genome, and three (Ghi_A05G04011, Ghi_A07G00666, and Ghi_A08G15006) and two (Ghi_D05G01481 and Ghi_D07G00601) orthologous genes in *G. hirsutum* (AD1) A and D genomes, respectively (Figure 3A). AT3G02990.1 and AT5G16820.1 (belonged to HSFA) have one orthologous gene (Gar02G15260) in *G. arboreum* (A2) genome, three orthologous genes (D5.v1.pred_00011647-RA, D5.v1.pred_00020752-RA, and D5.v1.pred_00039203-RA) in *G. raimondii* (D5) genome, and three (Ghi_A02G08261, Ghi_A08G13706, and Ghi_A13G10686) and three (Ghi_D03G03196, Ghi_D08G12936, and Ghi_D13G09616) orthologous genes in *G. hirsutum* (AD1) A and D genomes, respectively (Figure 3B). This result indicated that the HSF gene family have experienced independent evolution after divergence from *A. thaliana* and the ancestor of cotton species. *A. thaliana* HSF gene (AT4G36990.1, belonged to HSFB type), two genes (Gar02G18780 and Gar10G10520) in *G. raimondii* (D5) genome, three genes (D5.v1.pred_00008272-RA, D5.v1.pred_00014308-RA, and D5.v1.pred_00038859-RA) in *G. raimondii* (D5) genome, and three (Ghi_A02G09141, Ghi_A10G05501, and Ghi_A11G02666) and three (Ghi_D03G01096, Ghi_D10G04861, and Ghi_D11G02981) genes in *G. hirsutum* (AD1) A and D genomes were clustered together and divided into two groups following the divergence from the common ancestor of *A. thaliana* and *Gossypium* lineage (Figure 3C). In *A. thaliana* genome, only one gene (AT3G24520.1) was identified to belong to HSFC. Through the analysis of phylogeny, AT3G24520.1 has one orthologous gene (Ghe06G07640) in *G. herbaceum* (A1) genome, three orthologous genes (Gar05G02340, Gar06G07660, and Gar08G30890) in *G. arboreum* (A2) genome, three orthologous genes (D5.v1.pred_00000276-RA, D5.v1.pred_00011882-RA, and D5.v1.pred_00031281-RA) in *G. raimondii* (D5) genome, and three (Ghi_A05G03651, Ghi_A06G03346, and Ghi_A08G14781) and three (Ghi_D05G01101, Ghi_D06G03211, and Ghi_D08G13996) in *G. hirsutum* (AD1) A and D genomes, respectively (Figure 3D).

### Evolution of HSF Genes in Upland Cotton

To trace the evolutionary history of HSF genes in the Upland cotton genome, we combined the MCscanX software and validation of phylogeny to confirm HSF orthologous gene pairs between *G. herbaceum* (A1), *G. arboreum* (A2), and *G. hirsutum* (AD1) A genomes and between *G. raimondii* (D5) and *G. hirsutum* (AD1) D genomes. This result will help to trace the evolution of HSF genes in *G. hirsutum* genome clearly. In *G. hirsutum* (AD1) genome, 78 HSF genes were identified, namely 40 and 38 HSF genes distributed into A and D genomes, respectively. From the comparisons between *G. herbaceum* (A1), *G. arboreum* (A2), and *G. hirsutum* (AD1) A genomes, we got 11 and 64 all-against-all HSF gene pairs, respectively (Supplementary File 2). Combined with the phylogeny of *A. thaliana*, *G. herbaceum* (A1), *G. arboreum* (A2), *G. raimondii* (D5), and *G. hirsutum* (AD1), four HSF genes in *G. herbaceum* (A1) genome were investigated to get four HSF orthologous genes in *G. hirsutum* (AD1) A genome, and 17 HSF genes in *G. arboreum* (A2) genome and 17 HSF orthologous genes in *G. hirsutum* (AD1) A genome (Table 3) were obtained. This meant that only 17 HSF genes in *G. hirsutum* (AD1) A genome were detected to have orthologous genes in *G. herbaceum* (A1) and *G. arboreum* (A2) genomes, which represented 42.5% of the total HSF genes in *G. hirsutum* (AD1) A genome. A previous study indicated that all existing A genomes in *Gossypium* lineage originated from a common progenitor, A0 genome, and *G. herbaceum* (A1) and *G. arboreum* (A2) genome experienced independent evolution after the split of the A0 genome (Huang et al., 2020). Following this evolutionary relationship among different cotton genomes, these four collinear HSF genes between *G. herbaceum* (A1), *G. arboreum* (A2), and *G. hirsutum* (AD1) A genome existed in ancestral genome A0 and remained in *G. herbaceum* (A1), *G. arboreum* (A2), and *G. hirsutum* (AD1) A genomes although they experienced independently long-term evolutionary history, but HSF genes in *G. herbaceum* (A1) genome missed more according to orthologous analysis between *G. herbaceum* (A1), *G. arboreum* (A2), and *G. hirsutum* (AD1) A genomes.

**TABLE 3:** Statistics of HSF orthologous gene pairs between *G. herbaceum* (A1), *G. arboreum* (A2), *G. raimondii* (D5), and *G. hirsutum* (AD1) genomes.

Category	*G. hirsutum* (AD1)	*G. herbaceum* (A1)	*G. arboreum* (A2)	*G. raimondii* (D5)
A genome	Ghi_A01G06131		Gar01G15650	
	Ghi_A02G06021	Ghe02G13080	Gar02G12440	
	Ghi_A02G08261		Gar02G15260	
	Ghi_A02G09141		Gar02G18780	
	Ghi_A03G01196		Gar01G28870	
	Ghi_A03G05016		Gar03G17300	
	Ghi_A05G03651		Gar05G02340	
	Ghi_A05G04011	Ghe05G03320	Gar05G03090	
	Ghi_A05G12466		Gar05G21450	
	Ghi_A05G22286		Gar04G04540	
	Ghi_A06G03346	Ghe06G07640	Gar06G07660	
	Ghi_A08G14781		Gar08G30890	
	Ghi_A08G15006	Ghe08G31530	Gar08G31350	
	Ghi_A09G08111		Gar09G17530	
	Ghi_A10G05501		Gar10G10520	
	Ghi_A10G07266		Gar10G18400	
	Ghi_A11G00561		Gar11G01050	
D genome	Ghi_D01G05506			D5.v1.pred_00023184-RA
	Ghi_D02G04501			D5.v1.pred_00026581-RA
	Ghi_D03G01096			D5.v1.pred_00038859-RA
	Ghi_D03G03196			D5.v1.pred_00039203-RA
	Ghi_D03G04351			D5.v1.pred_00039503-RA
	Ghi_D03G09056			D5.v1.pred_00040456-RA
	Ghi_D03G10101			D5.v1.pred_00040705-RA
	Ghi_D05G01101			D5.v1.pred_00000276-RA
	Ghi_D05G01481			D5.v1.pred_00000361-RA
	Ghi_D06G00816			D5.v1.pred_00030721-RA
	Ghi_D06G03211			D5.v1.pred_00031281-RA
	Ghi_D06G12056			D5.v1.pred_00033238-RA
	Ghi_D07G00601			D5.v1.pred_00033482-RA
	Ghi_D08G03436			D5.v1.pred_00009636-RA
	Ghi_D08G10626			D5.v1.pred_00011142-RA
	Ghi_D08G12936			D5.v1.pred_00011647-RA
	Ghi_D08G13996			D5.v1.pred_00011882-RA
	Ghi_D09G04386			D5.v1.pred_00028656-RA
	Ghi_D09G06176			D5.v1.pred_00029020-RA
	Ghi_D09G07796			D5.v1.pred_00029359-RA
	Ghi_D09G09531			D5.v1.pred_00030009-RA
	Ghi_D09G10346			D5.v1.pred_00030184-RA
	Ghi_D10G01611			D5.v1.pred_00014981-RA
	Ghi_D10G04861			D5.v1.pred_00014308-RA
	Ghi_D10G08231			D5.v1.pred_00013550-RA
	Ghi_D11G00486			D5.v1.pred_00008670-RA
	Ghi_D11G02981			D5.v1.pred_00008272-RA
	Ghi_D11G06781			D5.v1.pred_00007473-RA
	Ghi_D12G12826			D5.v1.pred_00016131-RA
	Ghi_D12G13776			D5.v1.pred_00015928-RA
	Ghi_D13G01336			D5.v1.pred_00018925-RA
	Ghi_D13G09616			D5.v1.pred_00020752-RA
	Ghi_D13G11381			D5.v1.pred_00021132-RA

In *G. hirsutum* (AD1) D genome, 33 of 38 HSF genes were detected to have orthologous genes in *G. raimondii* (D5) genome, representing 86.8% of the total HSF genes in *G. hirsutum* (AD1) D genome. For *G. raimondii* (D5) genome, all HSF genes were detected to have orthologous genes in *G. hirsutum* (AD1) D genome. These results meant that members of the HSF gene family in *G. raimondii* (D5) genome and *G. hirsutum* (AD1) D genome kept excellent collinear relationships after the formation of *G. hirsutum* (AD1), but HSF genes in *G. hirsutum* (AD1) D genome showed significant expansion compared with those in *G. raimondii* (D5) genome.

### Analysis of Tandem Duplication Events of HSF Genes in Cotton Genomes

In this part, we investigated this important mechanism that occurred in different cotton genomes to get more evidence for the expansion of the HSF gene family. With the method implemented in PTGBase, we obtained 5,672, 5,118, 6,237, 6,013, 4,956, 8,070, 5,630, 4,930, 7,635, 25,877, 26,570, 26,306, and 21,726 tandemly duplicated genes in *G. herbaceum*, *G. arboreum*, *G. thurberi*, *G. raimondii*, *G. turneri*, *G. longicalyx*, *G. australe*, *G. kirkii*, *G. hirsutum*, *G. barbadense*, *G. tomentosum*, *G. mustelinum*, and *G. darwinii*, representing 12.9, 11.83, 19.79, 14.76, 12.49, 21.03, 14.71, 13.44, 10.27, 34.66, 33.94, 35.23, and 27.75% of total protein-coding genes in *G. herbaceum*, *G. arboreum*, *G. thurberi*, *G. raimondii*, *G. turneri*, *G. longicalyx*, *G. australe*, *G. kirkii*, *G. hirsutum*, *G. barbadense*, *G. tomentosum*, *G. mustelinum*, and *G. darwinii* genomes (Table 4). Through investigation of 621 HSF genes among 13 cotton genomes, only two tandemly duplicated genes (Gothu.00002342-RA and Gothu.00002341-RA) distributed into one tandem array in *G. thurberi* (D1) genome were identified following the identification of tandemly duplicated genes in plants. However, for the remaining 12 cotton genomes, we did not get any tandemly duplicated genes of the HSF gene family in these cotton genomes. So, the TD events brought an increase of the members of the HSF gene family in *G. thurberi* (D1) genome, but the HSF gene family in 12 cotton species did not experience TD event, which meant that members of the HSF gene family were discretely distributed into 12 cotton genomes.

**TABLE 4:** Statistics of HSF tandemly duplicated genes in 13 cotton genomes.

Species	Karyotype	Total predicted genes	Numbers of total TD genes	Percentage (%)	Total HSF genes	Numbers of HSF TD genes
*G. herbaceum*	A1	43,952	5,672	12.9	4	0
*G. arboreum*	A2	43,278	5,118	11.83	17	0
*G. thurberi*	D1	31,520	6,237	19.79	29	2
*G. raimondii*	D5	40,743	6,013	14.76	33	0
*G. turneri*	D10	39,692	4,956	12.49	36	0
*G. longicalyx*	F1	38,378	8,070	21.03	38	0
*G. australe*	G2	38,281	5,630	14.71	36	0
*G. kirkii*	K	36,680	4,930	13.44	29	0
*G. hirsutum*	AD1	74,350	7,635	10.27	78	0
*G. barbadense*	AD2	74,651	25,877	34.66	78	0
*G. tomentosum*	AD3	78,281	26,570	33.94	84	0
*G. mustelinum*	AD4	74,660	26,306	35.23	79	0
*G. darwinii*	AD5	78,303	21,726	27.75	80	0

*TD, tandemly duplicated.*

### Expression Analysis of HSF Genes in *G. hirsutum* (AD1) and *G. barbadense* (AD2)

To investigate the expression differences of cotton HSF genes for heat stress tolerance, we analyzed the expression profiling of HSF genes in leaves under different treatments (control, heat, and cold) of *G. hirsutum* (AD1) acc. TM-1 and *G. barbadense* (AD2) acc. Hai7124. These treatments of different periods (hours) include control (0, 1, 3, 6, 12, 24 h), heat (1, 3, 6, 12, 24 h), and cold (1, 3, 6, 12, 24 h) in *G. hirsutum* (AD1) and *G. barbadense* (AD2), respectively. After curation, we got 32 treatments in total. Using all available RNA-seq short-reads data of different samples of *G. hirsutum* (AD1) and *G. barbadense* (AD2), we implemented an expression analysis of total protein-coding genes in *G. hirsutum* (AD1) and *G. barbadense* (AD2) genome and further retrieved expression values of 78 and 78 HSF genes from different *G. hirsutum* (AD1) and *G. barbadense* (AD2) samples, respectively (Supplementary File 3). Out of 78 HSF genes in *G. hirsutum* (AD1) genome, 76 HSF genes were detected to have expression in 16 samples, with 2 HSF genes (Ghi_A01G06131 and Ghi_D01G05506) having no expression in any of the samples. In *G. barbadense* (AD2) genome, 76 HSF genes have expression among 16 samples and two HSF genes (Gobar.A01G125400.1 and Gobar.D01G132500.1) were not detected to have expression in any of the samples. In *G. hirsutum* (AD1) genome, the 76 expressed HSF genes were divided into two different groups, I and II, and the I group contained 35 HSF genes, with the II group containing 41 HSF genes (Supplementary Figure 1A). In *G. barbadense* (AD2) genome, 76 expressed HSF genes also were divided into two different groups, I and II, but the I group contained 46 HSF genes and the II group contained 30 HSF genes (Supplementary Figure 1B). These results revealed that the expressed HSF genes in *G. hirsutum* (AD1) and *G. barbadense* (AD2) indicated different expression patterns despite the implementation of identical experimental materials with identical heat and cold stresses.

**FIGURE 4:**
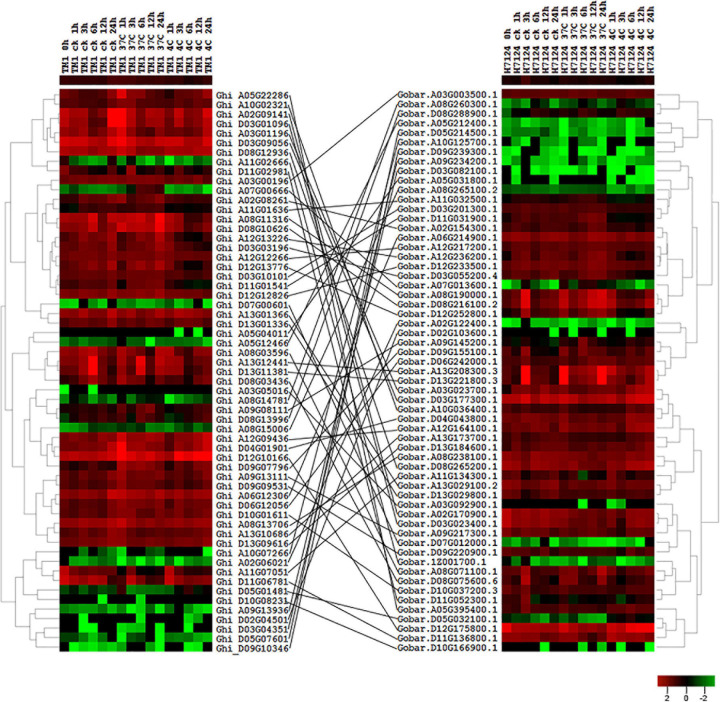
Expression profile of HSF orthologous genes between *G. hirsutum* (AD1) and *G. barbadense* (AD2) genomes. The black line represents the collinear relationships of HSF orthologous genes between *G. hirsutum* (AD1) and *G. barbadense* (AD2) genomes.

**FIGURE 5:**
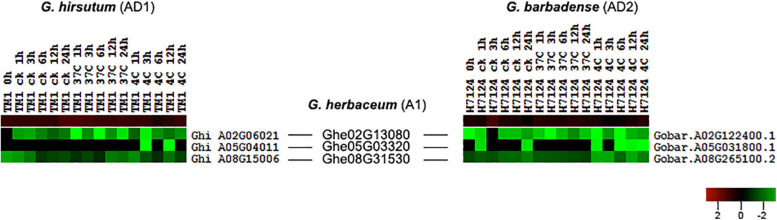
Expression difference of three HSF orthologous genes in *G. hirsutum* (AD1) and *G. barbadense* (AD2) genomes compared with *G. herbaceum* (A1) genome. The black line represents the collinear relationships of HSF orthologous genes among *G. herbaceum* (A1), *G. hirsutum* (AD1), and *G. barbadense* (AD2) genomes.

According to the collinear relationship between *G. hirsutum* (AD1) and *G. barbadense* (AD2), we obtained 61 HSF orthologous gene pairs between the two genomes. In *G. hirsutum* (AD1) genome, 31 and 30 HSF genes from 61 orthologous gene pairs were located on *G. hirsutum* (AD1) A and D genomes, respectively, with one HSF gene locating on unknown pseudomolecular in *G. hirsutum* (AD1) D genome. In *G. barbadense* (AD2) genome, there were 61 HSF genes from 61 orthologous gene pairs and one HSF gene was not detected on any chromosome, with 30 HSF genes locating on A genome and 30 HSF genes locating on D genome. The no expression HSF genes, Ghi_A01G06131 versus Gobar.A01G125400.1 and Ghi_D01G05506 versus Gobar.D01G132500.1, were orthologous gene pairs between *G. hirsutum* (AD1) and *G. barbadense* (AD2) genomes. After removing the no expression HSF genes, 59 HSF orthologous gene pairs between *G. hirsutum* (AD1) and *G. barbadense* (AD2) genomes were selected to investigate the difference of expression patterns (Figure 4). Through comparative analysis among *G. herbaceum* (A1), *G. hirsutum* (AD1), and *G. barbadense* (AD2), four HSF genes in *G. herbaceum* (A1) were detected to have four and four orthologous HSF genes in *G. hirsutum* (AD1) and *G. barbadense* (AD2), respectively. Only three of four HSF orthologous gene pairs between *G. hirsutum* (AD1) (Ghi_A02G06021, Ghi_A05G04011, and Ghi_A08G15006) and *G. barbadense* (AD2) (Gobar.A02G122400.1, Gobar.A05G031800.1, and Gobar.A08G265100.2) were expressed in different abiotic stress treatments and demonstrated different downregulated expression patterns regardless of control, cold, or heat stresses (Figure 5).

## Discussion

**FIGURE 6:**
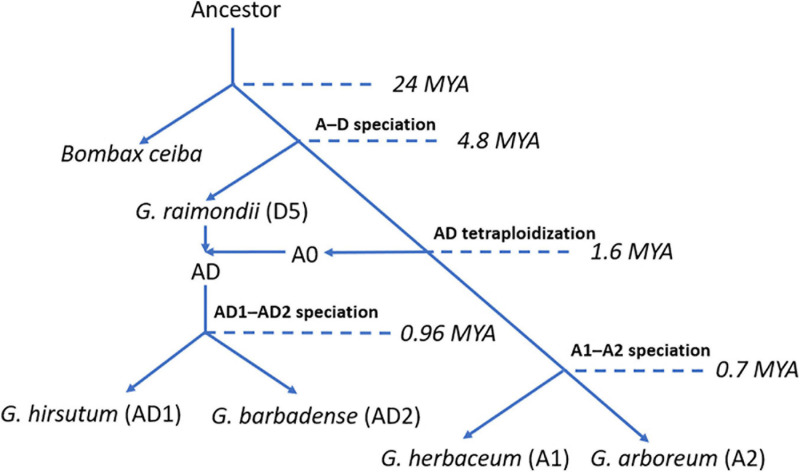
The evolution of the cotton genomes.

To trace the evolutionary history of HSF genes in *G. hirsutum* (AD1) genome, we collected five genome-sequenced plant species, including the model plant *A. thaliana*, three cotton diploid species [*G. herbaceum* (A1), *G. arboreum* (A2), and *G. raimondii* (D5)] and one tetraploid species [*G. hirsutum* (AD1)]. The allotetraploid cotton, *G. hirsutum* (AD1), named Upland cotton, is one of the most economically important crops which dominates over 98% of cotton production worldwide. Previous reports illustrated that *G. hirsutum* (AD1) was generated from hybridization between cotton A-genome progenitor and D-genome ancestor by chromosome doubling. Upland cotton D genome originated from *G. raimondii* (D5), and the A genome originated from the common progenitor, A0 genome, of all existing A genomes in *Gossypium* lineage. Furthermore, the formation of *G. hirsutum* (AD1) has preceded the split of *G. herbaceum* (A1) and *G. arboreum* (A2). These two cotton A genomes were shown to evolve independently from their common ancestor (Figure 6; Huang et al., 2020). Through the identification of HSF genes in *G. hirsutum* (AD1) genome, we got 40 and 38 HSF genes in *G. hirsutum* (AD1) A and D genomes, which meant that the D genome in *G. hirsutum* (AD1) missed two HSF genes compared with *G. hirsutum* (AD1) A genome. Through comparative genomic analysis between *G. raimondii* (D5) and *G. hirsutum* (AD1) D genome, we got 33 HSF orthologous gene pairs between the two genomes, which meant that *G. raimondii* (D5) missed five HSF genes after independent evolution. For the comparisons of *G. herbaceum* (A1), *G. arboreum* (A2), and *G. hirsutum* (AD1) A genomes, we got 17 HSF orthologous gene pairs between *G. arboreum* (A2) and *G. hirsutum* (AD1) A genomes, containing four HSF orthologous gene pairs, which meant that *G. herbaceum* (A1) and *G. arboreum* (A2) missed more HSF genes after the split of their common ancestor, but they kept four HSF orthologous genes inherited from their common ancestor, A0 genome. These four common conserved HSF genes in *G. herbaceum* (A1), *G. arboreum* (A2), and *G. hirsutum* (AD1) A genome might experience stronger natural selection pressures and performed a key biological function for resisting heat stress tolerance for adapting to the changing environment. From the evolutionary history of cotton diploid and tetraploid genomes, we knew that ancestral cotton genomes experienced loss of the members of the HSF gene family, but the current cotton tetraploid genomes kept more members of the HSF gene family.

Heat shock transcription factor gene family analysis among different cotton diploid and tetraploid genomes showed significant differences in number variation of the members of the HSF gene family. Apparently, members of the HSF gene family in cotton tetraploid genomes indicated a larger family size of the HSF gene family than those in cotton diploid genomes. Members of the HSF gene family in subgenomes of cotton tetraploid genomes showed expansion compared with those in cotton diploid genomes. These results demonstrated that HSF genes in cotton diploid genomes experienced weaker selection pressures leading to fast loss of HSF genes during the evolutionary history, but the HSF genes in subgenomes of cotton tetraploid genomes experienced stronger selection pressures after hybridization. The analysis of number variation of the members of the HSF gene family illustrated that the cotton tetraploid species may indicate stronger ability for the resistance of heat stress tolerance than diploid species.

A previous study illustrated that *G. hirsutum* (AD1) and *G. barbadense* (AD2) diverged after the formation of cotton allotetraploid AD genome at approximately 0.96 Mya, suggesting that these two genomes kept many orthologous gene pairs (Liu et al., 2015). Based on the conserved domain of HSF genes, we got 78 and 78 HSF genes in *G. hirsutum* (AD1) and *G. barbadense* (AD2) genomes, respectively. From collinear analysis, 122 HSF genes from 61 orthologous gene pairs in *G. hirsutum* (AD1) and *G. barbadense* (AD2) were used to detect the expression pattern of HSF orthologous genes in cotton tetraploid species. After the analysis of difference expression, 61 and 61 HSF genes in *G. hirsutum* (AD1) and *G. barbadense* (AD2), respectively, indicated totally different expression pattern, which meant the functional divergence of HSF orthologous genes between the two genomes. These results provided novel insights for functional studies and molecular breeding for the cotton community.

## Conclusion

According to the conserved domains of HSF gene family in plants, we identified 621 HSF genes among 13 genome-sequenced cotton species distributed into eight diploid and five tetraploid species. Comparative genomics analysis demonstrated that members of the HSF gene family in cotton diploid genome showed a significant expansion in *G. thurberi* (D1), *G. raimondii* (D5), *G. turneri* (D10), *G. longicalyx* (F1), *G. australe* (G2), and *G. kirkii* (K) compared with those in *G. herbaceum* (A1) and *G. arboreum* (A2). As expected, cotton tetraploid genomes have more members of the HSF gene family than diploid genomes. Phylogenetic analysis revealed that 621 HSF genes in 13 cotton genomes were clustered into two different groups, and the I group contained all HSF genes of HSFA and HSFC, with the II group containing all HSF genes of HSFB according to the conserved domains and phylogeny of HSF genes. Comparing *A. thaliana*, *G. herbaceum* (A1), *G. arboreum* (A2), *G. raimondii* (D5), *G. herbaceum* (A1), and *G. arboreum* (A2), four HSF genes were inherited from the common progenitor, A0, of all existing cotton A genomes, and members of the HSF gene family indicated a significant expansion in *G. arboreum* (A2) and *G. hirsutum* (AD1) A genome compared with *G. herbaceum* (A1). However, HSF genes in *G. hirsutum* (AD1) D genome experienced a significant expansion compared with those in *G. raimondii* (D5). Analysis of TD events of HSF genes revealed that the HSF gene family in *G. thurberi* (D1) genome has experienced TD event, but not in the remaining 12 cotton genomes. Expression analysis revealed that HSF orthologous gene pairs between *G. thurberi* (D1) and *G. barbadense* (AD2) showed a totally different expression trend under identical heat and cold stresses, which is beneficial for cotton molecular breeding and directed screening. This study is the first to trace the evolutionary history and expression features of the HSF gene family in 13 cotton genomes and provides more biological evidence to study the HSF gene family in response to abiotic stress tolerance in cotton genomes, particularly expression diversity of HSF genes in different treatments of leaves among different *G. hirsutum* (AD1) and *G. barbadense* (AD2) accessions. We hope this work will provide novel insights for the study of HSF genes under environmental stress in different cotton genomes and a widespread application model for the study of HSF gene families in plants.

## Data Availability Statement

The original contributions presented in the study are included in the article/Supplementary Material, further inquiries can be directed to the corresponding author/s.

## Author Contributions

YL and QC conceived and designed the experiments. YL analyzed the data and prepared the manuscript. JW and JZ performed the experiments and analyzed the data. ZG performed phylogenetic analysis and revised the manuscript draft. ZL collected genomic data, performed comparative analysis of HSF genes, and revised the manuscript draft. XA and XL performed the tandem duplication analysis of HSF genes and revised the manuscript draft. QC revised the manuscript. All authors read and approved the final manuscript.

## Conflict of Interest

ZL was employed by company Adsen Biotechnology Corporation. The remaining authors declare that the research was conducted in the absence of any commercial or financial relationships that could be construed as a potential conflict of interest.

## Footnotes

1 https://www.cottongen.org/

2 https://www.arabidopsis.org/download/index-auto.jsp?dir=/download_files/Sequences/Araport11_blastsets

3 http://pfam.xfam.org/

## Supplementary Material

Supplementary Figure 1 **(A,B)** represent the expression heatmap of HSF genes in *G. hirsutum* (AD1) and *G. barbadense* (AD2) genomes, respectively. I and II represent the different clusters of the expressed HSF genes in *G. hirsutum* (AD1) and *G. barbadense* (AD2) genomes, respectively.

Supplementary File 1 Gene basic information of HSF genes in *A. thaliana* and 13 cotton genomes.

Supplementary File 2 Collinear analysis between *G. herbaceum* (A1), *G. arboreum* (A2) and *G. hirsutum* (AD1) A genomes, as well as between in *G. raimondii* (D5) genome and *G. hirsutum* (AD1) D genome. A1-AD1A.collinearity sheet represented for the collinear gene pairs between *G. herbaceum* (A1) and *G. hirsutum* (AD1) A genomes; A2-AD1A.collinearity sheet represented for the collinear gene pairs between *G. arboreum* (A2) and *G. hirsutum* (AD1) A genomes; and D5-AD1D.collinearity sheet represented for the collinear gene pairs between in *G. raimondii* (D5) genome and *G. hirsutum* (AD1) D genome.

Supplementary File 3 Expression values of HSF genes in *G. hirsutum* (AD1) acc. TM-1 and *G. barbadense* (AD2) acc. Hai7124. Expression of HSFs in TM1 sheet represented for expression values of HSF genes in *G. hirsutum* (AD1) acc. TM-1 and Expression of HSFs in Hai7124 sheet represented for expression values of HSF genes in *G. barbadense* (AD2) acc. Hai7124.

## Abbreviations

HSF: heat shock transcription factor
HSP: heat shock protein
DBD: DNA-binding domain
OD: oligomerization domain
NLS: nuclear localization signals
NES: nuclear export signals
AHA: activator peptide proteins
RD: repressor domains
TD: tandem duplication
Mya: million years ago
HMM: hidden Markov model
ML: maximum likelihood
