# Supplementary figures and images for: Genome-Wide Comparative Analysis of Heat Shock Transcription Factors Provides Novel Insights for Evolutionary History and Expression Characterization in Cotton Diploid and Tetraploid Genomes
Source: Front Genet. 2021 Jun 8;12:658847. doi: 10.3389/fgene.2021.658847 (PMC8217870; doi:10.3389/fgene.2021.658847)

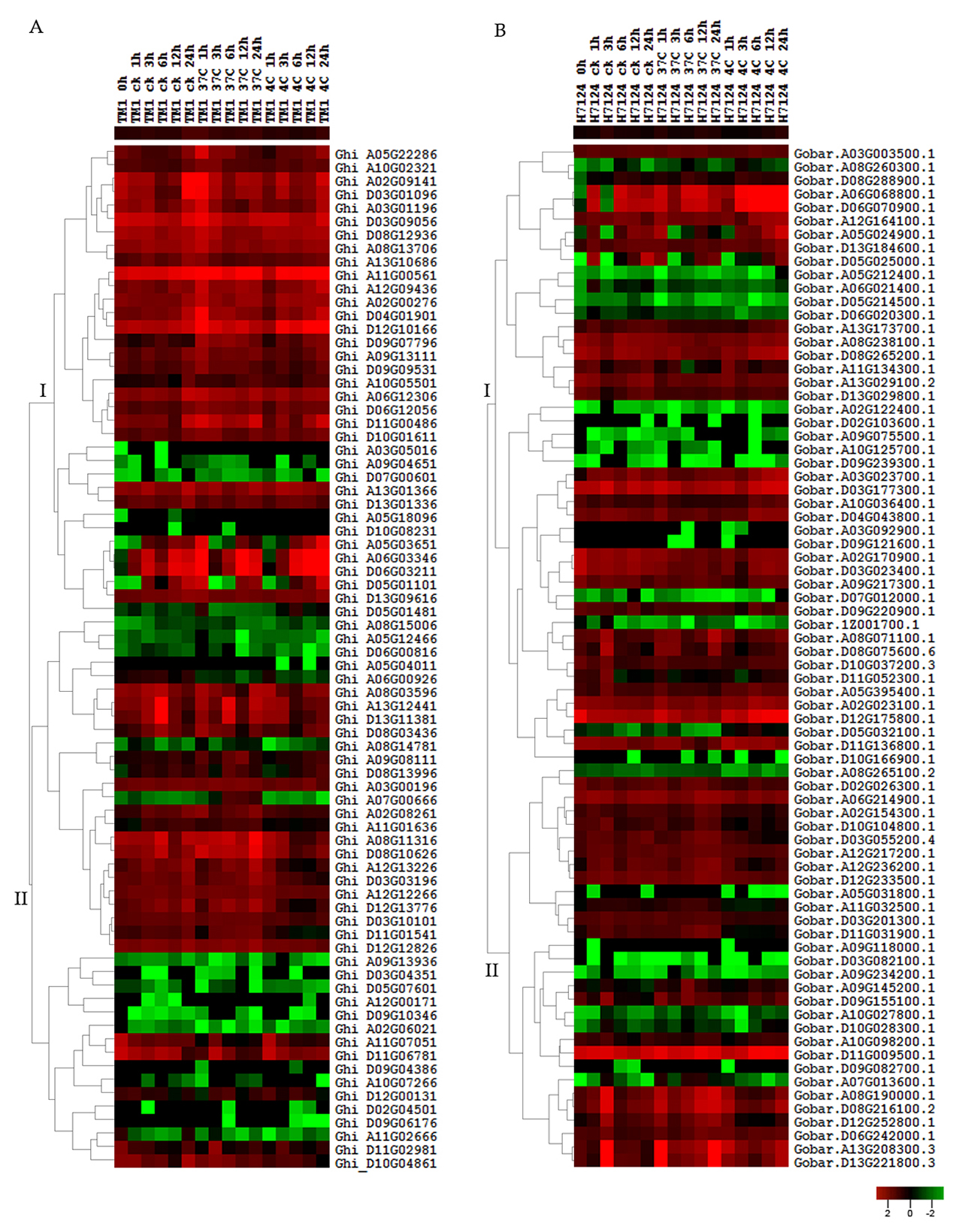

Supplement: Supplementary Figure 1 — (A,B) represent the expression heatmap of HSF genes in G. hirsutum (AD1) and G. barbadense (AD2) genomes, respectively. I and II represent the different clusters of the expressed HSF genes in G. hirsutum (AD1) and G. barbadense (AD2) genomes, respectively. [file Image_1.JPEG]
